# The role of nonfinancial factors in the Congressional Budget Office’s health insurance coverage projections

**DOI:** 10.1073/pnas.2525991123

**Published:** 2026-07-06

**Authors:** Nianyi Hong, Ben Hopkins

**Affiliations:** ^a^Congressional Budget, Health Analysis Division, Health Insurance Modeling Unit, Washington, DC 20515

**Keywords:** simulation modeling, nonfinancial factors, health insurance, ACA marketplace, Medicaid

## Abstract

Nonfinancial factors play an important role in people’s coverage decisions as premiums, cost sharing, and other factors that determine the financial value of health insurance cannot fully explain patterns in take-up rates. The Congressional Budget Office (CBO) is tasked with estimating the budgetary and coverage effects of policy and economic changes that affect health insurance markets, which includes considering the interactions between nonfinancial factors and policy design. The agency groups the nonfinancial factors it considers into three categories: ease of enrollment, awareness, and attitudes. Many recent policies have affected nonfinancial factors related to the take-up of Medicaid and marketplace insurance, such as the introduction of work requirements for Medicaid and access to zero-premium marketplace plans. This article explains how nonfinancial factors are handled and incorporated into CBO’s baseline health insurance projections and cost estimates, highlights the recent literature on the effect of these nonfinancial factors on insurance take-up decisions, and suggests future areas of research.

One way research influences policy is through its incorporation into the cost estimates and baseline projections produced by the Congressional Budget Office (CBO). The CBO is an agency that provides objective, nonpartisan information to support the Congressional budget process and to help Congress make effective budget and economic policy by offering technical assistance and preparing cost estimates for proposed legislation (1). To do so, CBO’s staff develops economic models that incorporate financial incentives and eligibility rules. The effects of policy on program participation and federal spending, however, depend not only on those financial factors but also on whether individuals are aware of public programs, want to enroll, and can easily do so. Accordingly, CBO’s models often incorporate nonfinancial factors to capture those behavioral responses.

In this article, we provide insight into how research on nonfinancial factors influences the legislative process by highlighting their role in CBO’s cost estimates related to health insurance coverage for the nonelderly population, with a focus on policy related to the Affordable Care Act. CBO publishes baseline projections of health insurance coverage and net federal subsidies for health insurance most years and frequently estimates the effect of legislation related to health insurance coverage that the Congress is considering (2).^*^ In its baseline projections published June 2024, CBO projected that the federal government would spend $27.5 trillion to subsidize health insurance coverage between 2025 and 2034. In those projections, about 10% of this spending (or $2.7 trillion) was to subsidize the coverage of an annual average of 13.5 million adults made eligible for Medicaid coverage by the Affordable Care Act (ACA) and 14.7 million subsidized enrollees in the health insurance marketplaces established by the ACA (3). The passage of the ACA, enacted in March 2010, made significant changes in federal programs and tax policies that affected the affordability and accessibility of insurance (4). The ACA gave states the option to expand eligibility for Medicaid—the main source of health insurance coverage for Americans who have very low income (5). It also established health insurance marketplaces (ACA marketplaces) where people can purchase nongroup health insurance coverage;^†^ some enrollees qualify for subsidies toward the purchase of that coverage (3).

Since its passage, the ACA has been amended several times, most recently by the 2025 Reconciliation Act. Those policies have affected health insurance coverage and the federal budget not only through their changes to who is eligible for subsidized health insurance but through their effects on take-up rates for Medicaid and ACA marketplace coverage. We define take-up rates in this context as the number of people who enroll in a program divided by the number of people who are eligible to do so. While financial factors, such as premiums and cost-sharing, are fundamental to the decision to enroll in health insurance, they do not fully explain patterns in take-up rates. Based solely on financial incentives, we would expect near-universal take-up of Medicaid and fully subsidized Marketplace plans. In reality, take-up is incomplete even among those with access to highly subsidized (6, 7)—or even free—health insurance with low cost-sharing. The number of people who enroll in health insurance coverage also depends on whether people are aware of it (“awareness”), whether they want to enroll in it (“attitudes”), and whether it is easy to do so (“ease of enrollment”).

When estimating the budgetary and coverage effects of health insurance policies, these nonfinancial factors are primary considerations. For Medicaid and subsidized marketplace coverage, take-up and retention generally require individuals to navigate multiple stages, including learning about the program, completing an initial eligibility determination, periodically reaffirming eligibility through renewals, and—in the case of the marketplaces—paying a premium. Nonfinancial factors can affect participation at each of these points and can be complex as different programs and states often have their own specific rules. These rules have major implications for the effects of legislation. For example, Section 71119 of the 2025 Reconciliation Act directs states to require certain Medicaid enrollees to satisfy “community engagement requirements.” The CBO estimated that about 2.9 million people in 2034 will lose coverage because they do not satisfy the requirements or qualify for an exception. It estimated that a further 2.8 million people in 2034—a nearly equal number—who satisfy the requirements or qualify for an exception will lose coverage because the requirements will add steps to the application process (8).

Most of the nonfinancial factors that affect whether people are aware of coverage options, whether they want to enroll in them, and whether it is easy to do so are closely tied to two broader aspects of the design of health insurance programs: administrative burdens and choice infrastructure. Administrative burdens, or the “frictions of interacting with government,” have been shown to reduce take-up of subsidized health insurance and other public programs (9–11). When intentional, administrative burdens are used to maintain program integrity or screen out individuals who do not value program participation but reduce take-up among untargeted groups as well (12). Burdens can also be imposed by parties other than the government, like health insurance issuers. Policies that alter administrative burdens or shift them from the individual to the state or federal government or a third-party, like enrollment assisters, can affect take-up (13).

Choice infrastructure, or the way that information is presented to consumers, also plays a crucial role in determining whether consumers take-up health insurance and enroll in a suitable plan. Health insurance is a complex and poorly understood product. 42% of individuals in one study found plan information difficult to understand and in another, 42% of individuals failed to correctly define what a deductible is, and 66% failed to correctly define coinsurance (14–16). This lack of knowledge can reduce take-up of health insurance coverage (17). A wide range of policies affect the structure of the choice environment, including those related to standardized plan designs, website design, nudges, decision support tools, and enrollment assistance (14, 18–21).

To explain how CBO incorporates nonfinancial factors in its coverage projections and estimates, we begin by discussing two models the agency uses to project the budgetary and coverage effects of health insurance policies and the role of nonfinancial factors therein: the agency’s health insurance microsimulation model, HISIM2, which is focused on modeling private insurance, and a model of Medicaid enrollment.^‡^ We then discuss recent research on topics related to the effect of policy on the take-up of subsidized coverage in Medicaid and through the ACA marketplaces.^§^ For each research topic area, we provide an overview of key findings, a sense of magnitude for effects on take-up rate, and an example of a CBO estimate related to the topic. Finally, we provide three examples of topics where research is lacking and would improve CBO’s modeling of health insurance coverage and cost estimates.

## Nonfinancial Factors in CBO’s Models

To provide insight into how CBO incorporates nonfinancial factors into its models, we describe two case studies. Our first case study discusses how nonfinancial factors are formally incorporated into the agency’s health insurance microsimulation model, HISIM2, which is focused on modeling private insurance. HISIM2 is not the only model that CBO uses to estimate the effects of health insurance policies. For some policies where existing models cannot be easily modified to capture nonfinancial factors that are integral to the policies’ effects—including many policies related to Medicaid—CBO crafts separate models that explicitly include nonfinancial factors. Our second case study briefly describes how CBO estimated the effects of changing the Medicaid eligibility redetermination period from 12 mo to 6 mo as per the 2025 Reconciliation Act.

### Case Study 1: CBO’s Health Insurance Simulation Model, HISIM2.

#### Model Background.

CBO uses its health insurance simulation model, HISIM2, to estimate the effects of legislative and economic changes on health insurance coverage and premiums, especially related to employer-based and nongroup health insurance. HISIM2 is a structural microsimulation model. The model first simulates households’ health insurance eligibility and the characteristics of their choices, e.g., whether they are eligible for premium tax credits (PTCs) and their expected out-of-pocket spending on premiums and health care if covered by a plan purchased through the ACA marketplaces.^¶^ It then predicts what type of health insurance coverage, if any, households enroll in. Because eligibility for and the attractiveness of private health insurance depends on the same factors for public health insurance, like Medicaid, HISIM2 models both. For example, the model can predict the effect of a change in Medicaid eligibility rules on eligibility for PTCs and demand for employment-based insurance.

In HISIM2, households choose between coverage options based on the expected utility they receive from each option.^#^ A household collectively chooses the type of health insurance coverage in which to enroll each of its members. The choice set of a health insurance unit (HIU) is restricted by the eligibility of its members for public insurance, subsidized marketplace insurance, and employment-based insurance. People within the same household may not be eligible for the same type of coverage and do not necessarily choose the same coverage option. Households make decisions by maximizing utility in a random utility model. Some parameters in the model are set based on CBO’s assessment of the research literature.

#### Role of Nonfinancial Factors in the Model.

The utility for an alternative partly depends on observable, financial factors like the household’s total income and out-of-pocket health care spending under the alternative.^‖^ It also depends on “alternative-specific constants” (ASCs) that represent the effect of average unobserved preferences across households for different types of insurance (22). The econometric interpretation of the ASCs is very similar to that of fixed effect estimates in a reduced form model. These terms capture the combined effect of nonfinancial factors on the attractiveness of different types of insurance coverage. In addition to nonfinancial factors, the ASCs capture unmodeled financial factors like access to health care (e.g., through network breadth) and the intrinsic value of that care and compensate for the model’s imperfect representation of reality. ASCs vary by alternative between single and multiperson households, by age and income for marketplace coverage, and between those made eligible for Medicaid by the Medicaid expansion and those who were not. Without these terms, HISIM2 would overestimate the importance of observable financial considerations in the health insurance decision.

The ASCs are calibrated such that, conditional on expected utility, the model’s estimated coverage totals match coverage targets from survey and administrative data sources. The ASCs are initially calibrated to the estimated coverage distribution across insurance types in the model’s base year. These targets are based on the best available survey and administrative data on health insurance coverage for each type and then reconciled so that enrollment in each type of coverage plus the number of uninsured sums to the total civilian noninstitutionalized population under 65. The ASCs are adjusted in later years to align HISIM2’s projections with the latest survey and administrative data on enrollment.

In years where data are not available, CBO consults related economic research to construct its estimates of the effects of nonfinancial factors on coverage and finds ASCs that reproduce those effects. Consider, for example, the effect of the scheduled expiration of the enhanced premium tax credits in the ACA marketplaces after 2025 on CBO’s coverage projections. In 2025, CBO calibrates the ASCs for marketplace coverage in HISIM2 to actual data on enrollment by age and income in that year. Thereafter, the model projects a decline in marketplace enrollment corresponding to the increase in out-of-pocket premiums for marketplace coverage. However, the agency also makes two adjustments to ASCs: a permanent downward adjustment to reflect the decreased availability of zero-premium silver plans and a temporary upward adjustment to reflect the effect of inertia on the decision to take up marketplace insurance.^**^

CBO embeds nonfinancial factors in HISIM2 rather than estimating their effects separately by adding them outside the model for several reasons. First, it allows CBO’s projections to capture the interactions between nonfinancial factors and financial factors that drive take-up, like premiums. Second, it allows the effects of nonfinancial factors to vary across groups with relatively high and low take-up rates. Finally, it allows nonfinancial factors to have spillovers on employer offers of insurance, the ACA risk pool, and substitute products like nongroup insurance purchased outside the ACA marketplaces.

### Case Study 2: CBO’s Medicaid Modeling.

#### Model Background.

CBO produces Medicaid cost estimates using an analytical framework that projects federal spending and enrollment over the 10-y budget window relative to its current-law baseline. Rather than relying on a single, fixed model, CBO tailors its approach to the specific proposal under consideration. This broad approach allows CBO to combine the sources of data and empirical evidence most relevant to a given policy change to assess how legislative proposals would affect key components of the Medicaid program, including eligibility, enrollment, service use, payment rates, and federal matching payments. Depending on the proposal, the analysis may incorporate administrative enrollment and spending data, survey data, demographic and economic projections, and detailed information about state Medicaid policies. CBO also incorporates historical experience to inform assumptions about behavioral responses by enrollees, states, and providers. The resulting projections estimate how legislation would change Medicaid enrollment and federal spending over time.

Federal proposals that specifically affect Medicaid coverage usually alter the eligibility requirements for Medicaid, the eligibility determination process, or the financing of the program and spur changes in state policies regarding the same. In evaluating such proposals, CBO considers how the policy would change Medicaid, and then an assessment of coverage gain or loss based on available data and historical precedents from prior Medicaid program changes, or when necessary, other low-income programs.

#### Role of Nonfinancial Factors in the Model.

One example of how CBO incorporates nonfinancial factors into Medicaid estimates is the proposal to change the eligibility redetermination period from 12 mo to 6 mo in the 2025 Reconciliation Act. Section 71107 of that act requires states to conduct Medicaid eligibility redeterminations every 6 mo for individuals made eligible in the ACA expansion, beginning in January 2027. Nonfinancial factors that affect ease of enrollment and awareness play a significant role in this policy, which makes no financial changes to obtaining coverage. Based on historical enrollment patterns, CBO estimates that approximately 10 percent of ACA expansion enrollees will lose coverage following the first 6-mo redetermination. This estimate is derived by modeling the probability of disenrollment using trends from standard 12-mo redetermination intervals and translating those to a more frequent review process. As a result, some people are expected to be removed from the program earlier than would have occurred before the 2025 reconciliation act became law. CBO expects that 70 percent of those who lose coverage will do so for procedural reasons such as missed paperwork, problems with communication, or difficulty navigating the reenrollment process and the remaining 30 percent will be determined ineligible under Medicaid’s criteria (23, 24).

## Nonfinancial Factors in the Academic Literature

Previous literature has established psychological factors that affect the decision to take-up health insurance. This literature is well surveyed (25, 26); however, the literature has expanded significantly since these survey papers were published as researchers study policies related to the ACA. In this section, we summarize and group some of the recent research on nonfinancial factors that has been particularly important for CBO’s work and used in CBO’s modeling. Rather than presenting a comprehensive literature review, we focus on research that has played a significant role in CBO’s estimates and is related to the populations made eligible for subsidized health insurance coverage by the ACA.

As mentioned previously, CBO has found it helpful to categorize the nonfinancial factors it considers into three groups: awareness, attitudes, and ease of enrollment. Put differently, these groups include factors that affect whether people know about a program, whether they want to enroll in it, and how difficult it is for them to do so. We present these factors in this order to roughly correspond with the order that an individual would go through to sign-up for health insurance coverage: They must be aware that the insurance option exists, be inclined to take it up, and finally, sign-up for coverage.

Within each of these categories, we further divide the literature by factor. Some of these factors correspond to a specific type of policy (e.g., automatic enrollment policies) and others to a more general factor (e.g., stigma against Medicaid). For each factor, we provide an overview of recent research, characterize the magnitude of its effect on take-up rates, and provide an example of where the factor has come up in CBO’s work.

CBO reviewed and assessed the empirical studies of the effect of nonfinancial factors on take-up and then categorized the magnitude of the take-up rate change as the result of each of these factors. Throughout this review, our focus is on changes in take-up rates rather than changes in the size of the eligible population. We note, however, that many empirical studies—particularly those using administrative data—define take-up using denominators that may differ from the true eligible population, either because eligibility is imperfectly observed or because the policy intervention itself alters renewal rules or screening processes. As a result, estimated effect sizes may not be directly comparable across studies.

In Table 1, we categorize the impact on take-up rate as very small (less than 1 percentage point change), small (between 1 to 5 percentage point change), moderate (between 5 to 10 percentage point change), or large (greater than 10 percentage point change). We caution that these categorizations are only intended to give a sense of the relative magnitude of effects found in the literature; the estimated effect of specific policies depends on the design of those policies and their interactions with concurrent policies and economic conditions, as well as any potential changes to who is eligible for subsidized coverage. In addition, many of these factors and policies (such as work requirements or zero-premium plans) have direct effects as well; the literature that we reference attempts to isolate the nonfinancial effects, but these additional factors make it difficult to provide more than a general range of magnitudes. Finally, the depth and consistency of the evidence vary across nonfinancial factors—having the greatest amount of evidence for policies that directly alter administrative processes or defaults and more limited for factors related to awareness and attitudes—but Table 1 is intended to summarize the direction and approximate magnitude of effects documented in the literature rather than to provide a formal assessment of strength of evidence.

**Table 1.** Evidence from published studies on the effect of nonfinancial factors of take-up of coverage through Medicaid and the ACA marketplaces

Category	Factor	Examples of literature	Magnitude of impact on take-up rate
Awareness	Welcome mat effects	Frean et al. (27), Hudson et al. (28), McMorrow et al. (29), Sonier et al. (30)	Small
	Advertising	Aizawa and Kim (31), Karaca-Mandic et al. (32)	Small
	Public enrollment assistance	Myerson and Li (33)	Very small
Attitudes	Immigration and chilling effects	Haley et al. (34), Sommers et al. (35), Twersky (36)	Large
	Individual mandate: Nonfinancial effects	Fiedler (37), Lurie et al. (38)	Very small
	Stigma	Celhay et al. (39)	Very small
Ease of enrollment	Automatic enrollment	Dague et al. (40), Fox et al. (41), Nelson et al. (42), Wolf et al. (43)	Large
	Work requirements	Folse et al. (44), Sommers et al. (45)	Large
	Zero-premium plans*	Abdus et al. (46), Drake and Anderson (47), McIntyre et al. (48)	Moderate

We categorize the impact on take-up rate as very small (less than 1 percentage point change), small (between 1 to 5 percentage point change), moderate (between 5 to 10 percentage point change), or large (greater than 10 percentage point change).

^*^A zero-premium plan is a health insurance plan that is fully subsidized either because enrollees are not required to pay a premium (as with most Medicaid programs) or because subsidies fully cover the plan’s premium.

### Awareness.

Many individuals eligible for Medicaid or subsidized marketplace plans remain uninsured because they are unaware of their eligibility for financial assistance and the deadlines to apply. The relationship between awareness and take-up is well established by a robust literature showing that informational nudges, like letters, texts, and phone calls, increase take-up (49, 50). Certain groups tend to have lower awareness of public programs than others, including people who are relatively healthy or do not speak English as their first language (49, 51).

#### Welcome Mat Effects.

In Medicaid, welcome mat effects occur when expansions of eligibility aimed at one group prompt eligible but unenrolled individuals in a different group to enroll due to heightened awareness or easier access. The ACA’s Medicaid expansions produced welcome mat effects among two notable groups. First, Medicaid enrollment increased among previously eligible children, often because parents who gained eligibility became aware that their children were also eligible (28, 30, 52). Second, Medicaid enrollment increased among previously eligible individuals in states that did not expand Medicaid without crowding out of coverage from other sources such as private insurance (27, 29). In that case, news of Medicaid expansions in other states may have prompted individuals to determine their own eligibility. In CBO’s assessment, welcome mat effects have a small impact on health insurance take-up and are incorporated in its Medicaid modeling.

#### Advertising.

Advertising is used to make people aware that they may be eligible for subsidized health insurance coverage. Several studies have found that publicly sponsored TV advertising increased enrollment in the early years of the ACA marketplaces (with potential spillover effects on Medicaid enrollment) and that privately sponsored advertisements affected insurer market shares but not overall market size (31, 32, 53). In particular, research finds that advertising is more potent in the ACA marketplaces than in markets for other consumer goods (31). Additional work is necessary to identify whether these effects extend to other media and to later years, now that the marketplaces have grown larger and more stable, but the existing evidence suggests that advertising spending yields small increases in the take-up of public programs. CBO has estimated the effects of expanded spending on advertising and outreach programs that promote insurance purchased through the ACA marketplaces (54).

#### Enrollment Assistance.

Enrollment assisters help people determine if they are eligible for subsidized health insurance coverage and enroll in it. Most enrollees in the ACA marketplaces are assisted by agents or brokers compensated through per-enrollee commissions paid by insurance companies. Enrollees may also receive assistance from publicly funded enrollment assisters, most notably from “Navigators.” (The Navigators program provides federal grants to organizations to conduct enrollment assistance, outreach and education, and consumer assistance. Unlike agents and brokers, Navigators may not recommend specific plans.)

Despite receiving significant funding under the Obama and Biden administrations, relatively few enrollees have been assisted by Navigators (55). However, one study found that cuts to Navigator grants during the first Trump administration had a significant effect on marketplace enrollment for low-income households and insured rates for some types of people, but not for overall marketplace or Medicaid enrollment (33). The existing evidence suggests that publicly funded enrollment assistance has a very small effect on take-up; more research is needed on the effect of enrollment assistance from private brokers. Changes in funding for the Navigators program affect CBO’s baseline health insurance projections (56).

### Attitudes.

Subjective attitudes toward health insurance in general or specific programs or policies may influence coverage decisions.

#### Chilling Effects.

Recent research has estimated whether immigration policy has “chilling effects” on the take-up of public benefits by eligible individuals. Researchers have hypothesized that some eligible people forego public benefits like Medicaid or CHIP out of fear that doing so will put the immigration status of themselves or someone in their household at risk (57, 58). For example, a study focusing on chilling effects from the public charge rule, under which immigrants can be denied legal permanent resident status if they are deemed likely to depend on the government for subsistence, reports that 9.6 percent of adults in immigrant families with children reported that they or someone in their family avoided enrolling in Medicaid or CHIP “for fear of risking future green card status.” (59). While the chilling effect is particularly predominant in Hispanic communities, which comprise a majority of unauthorized immigrants, this effect has been shown among Asian immigrants as well (60, 61). These and other estimates suggest that chilling effects have a large effect on health insurance take-up among those in households with immigrants (35, 36, 62–65). CBO included chilling effects as part of an estimate of the coverage effects on Medicaid of some of the policies within the 2025 Reconciliation Act on Medicaid coverage (66).

#### Individual Mandate.

The individual mandate—a requirement that most Americans purchase health insurance or pay a penalty—was anticipated to increase health insurance coverage through a response to the financial penalty itself and through a “taste for compliance,” or a preference to follow a legal requirement even in the absence of financial incentives. Recent evidence suggests that the individual mandate slightly increased health insurance coverage take-up (37). The magnitude of this effect was much less than CBO projected prior to the implementation of the ACA and its subsequent repeal, as CBO projected a significantly larger response to the individual mandate penalty than was ultimately observed (67). Research finds some evidence of a small taste for compliance and that consumers responded more to the certain, near-term cost of a premium than to the future-cost of a penalty which may or may not be enforced (38, 68). The differential response to premiums and penalties point to behavioral factors in how consumers evaluate different types of financial incentives, such as time preferences, salience, and lack of understanding. Overall, the evidence on the individual mandate suggests that it had a very small impact on the health insurance take-up rate. CBO incorporated these nonfinancial factors in its revised estimate of the effects of the individual mandate penalty in 2017 (69).

#### Stigma.

Stigma, defined as a certain level of disutility associated with participation in a program, may affect the take-up of Medicaid or marketplace subsidies. The lack of program participation due to stigma has been shown in various welfare programs such as Aid to Families with Dependent Children (AFDC) and Supplemental Nutritional Assistance Program (SNAP) (70, 71). It is often difficult to determine if stigma is demonstrated in program take-up, or if it is an issue in the underreporting of take-up in surveys, a known issue for programs such as Medicaid (72, 73).

Focusing on Medicaid, surveys have reported that Medicaid recipients perceive that they are treated worse by providers, but there is mixed evidence that this affects take-up (74–78). Earlier research suggests that stigma decreases Medicaid take-up (79); more recent findings after the implementation of the ACA have shown more favorable perceptions of the program (39, 80, 81). Finally, stigma may differ across groups, reflecting variation in public attitudes regarding the types of people who are deserving of Medicaid coverage and under what conditions (82). Overall, while earlier evidence suggests that there may have been stigma toward Medicaid previously, more recent evidence suggests that stigma currently has a very small effect on Medicaid take-up. Stigma is one of many factors included in CBO’s Medicaid modeling.

### Ease of Enrollment.

Consumers are more likely to enroll in Medicaid and subsidized marketplace insurance when it is easier to do so. In addition, individuals must periodically demonstrate their eligibility to determine if they still qualify for the subsidy. For subsidized marketplace enrollees and Medicaid enrollees in some states, they must also select a plan and pay a premium. Consumers are more likely to be determined eligible for coverage and subsidies and therefore enroll if it is easier to demonstrate their eligibility and when they better understand the choice they are making.

#### Automatic Enrollment and Reenrollment.

Many policies have been implemented to reduce the administrative burdens involved in determining eligibility or easing enrollment for Medicaid. These include presumptive eligibility, which allows hospitals or other trusted entities to temporarily enroll people in Medicaid pending full eligibility determinations; express lane eligibility and renewal, which allows states to infer Medicaid eligibility based on eligibility findings from other programs like SNAP or TANF; and “ex parte” renewals, a requirement that states attempt to confirm eligibility using information available to the state. There is evidence that these and other policies that transfer administrative burdens from the individual to the state have increased Medicaid take-up (41). However, attempts to reduce administrative burdens can have unintended consequences: When Indiana replaced in-person Medicaid caseworker assistance with online and phone systems in 2007, enrollment fell by 4 percent within a year and remained lower even after the system was eliminated, reflecting reduced personalized assistance, greater sensitivity to application errors, and technical glitches (83).

Medicaid enrollees frequently lose coverage when their eligibility is redetermined, as is periodically required. In one study of renewals in Wisconsin prior to the COVID-19 public health emergency, 20% of a cohort of new enrollees lost coverage at the renewal deadline, of which nearly half reenrolled within 12 mo (84). One report on the unwinding of the Medicaid continuous eligibility provision reported that 69% of those disenrolled from Medicaid during the redetermination process were terminated “for paperwork or procedural reasons,” rather than for directly being found ineligible, although this is an overestimate as individuals who were ineligible and declined to reapply would be included under “procedural reasons” (85, 86).^††^ Consistent with this pattern, a recent randomized field experiment found that providing targeted renewal assistance modestly increases Medicaid renewal rates, with larger effects for certain subgroups, underscoring the role of administrative burdens (87).

Some studies have also used state variation in the implementation of ex parte renewal policies to determine the effect of automatic renewal policies on program participation and churn (88). The evidence from these studies is mixed. One study using survey data found no association with overall Medicaid take-up rates, but two others using Transformed Medicaid Statistical Information System (T-MSIS) administrative data show that ex parte reviews reduce disenrollment and shorten coverage gaps among enrolled beneficiaries (42, 89, 90).

In the ACA marketplace, three studies of autoenrollment policies in California and Massachusetts found that the elimination of small administrative burdens were associated with large increases in the take-up of marketplace insurance (12, 91, 92). In California, another study found that autoenrollment policies increased the take-up of cost-sharing reduction subsidies far more than an informational nudge (43).^‡‡^

Considering the above literature, we conclude that automatic enrollment and reenrollment have a large impact on take-up for both Medicaid and nongroup coverage. Some of these nonfinancial factors were included in CBO’s estimate of the effects of several rules meant to streamline enrollment in Medicaid as part of the 2025 Reconciliation Act (8).

#### Work Requirements in Medicaid.

As part of the substantial changes in Medicaid in the recently enacted 2025 Reconciliation Act—Medicaid work requirements (also known as community engagement requirements) are scheduled to go into effect nationwide as early as 2027 (93). Work requirements affect Medicaid coverage in two ways: through eligibility restrictions (which directly decrease the number of individuals eligible for the program) and by reducing take-up among those who are eligible due to increased administrative burdens. There is little empirical evidence regarding the effects of work requirements on Medicaid enrollment—although many states received federal approval for work requirements, only Arkansas and Georgia have implemented them (44).

Between June 2018 and April 2019, Arkansas’ Medicaid program required expansion adults between the ages of 30 to 49 to file monthly online reports on their employment or other activities to maintain coverage. One group of researchers estimate that, while in effect, the work requirements led to a drop in Medicaid and marketplace coverage of over 10 percentage points for Arkansans ages 30-49, compared to other states and age groups (45). Research suggests that this coverage effect was partly driven by administrative burdens from difficulties using the system, lack of internet, and incorrect beliefs on qualification for the program (94).

In July 2023, Georgia created their Pathways program, which expanded Medicaid eligibility to previously ineligible individuals with income up to 100% of federal poverty limit who also satisfied other eligibility conditions, including a work requirement. The state had expected to enroll 25,000 individuals in the program’s first year but enrolled 4,300 (95). The state’s Medicaid agency attributed some of the shortfall to administrative burdens, and recommended simplifying the application process, particularly the reporting process for work requirements.

Based on this limited evidence, CBO concludes that work requirements in the Medicaid program reduce take-up by a large amount. These work requirement effects were included as part of CBO’s estimate of the effects of Medicaid community engagement requirements as part of the 2025 Reconciliation Act (8).

#### Zero-Premium Plans.

Recent empirical literature suggests that people with access to health insurance plans without a net premium are more likely to take-up health insurance. The effect of access to zero-premium plans exceeds what can be explained by the price elasticity of demand. Research primarily attributes the excess effects to the administrative burden of having to pay a premium. In Medicaid and CHIP, nominal premiums increase the probability of disenrollment and reduce take-up rates, caseload, and enrollment (46, 96–100). For nongroup coverage, federal subsidies come in the form of the premium tax credit (PTC)—a tax credit that eligible people can use to lower the out-of-pocket cost of their monthly premiums for health insurance coverage purchased through the ACA marketplaces. Some marketplace enrollees have access to “zero-premium plans” for which the PTC fully covers the premium. In the marketplaces, the practice of silver loading and enhanced subsidies have made zero-premium bronze and silver plans broadly available to low-income households and spurred research into the effect of access to zero-premium plans on take-up.^§§^ The availability of zero-premium plans has been shown to have increased take-up of marketplace insurance in California, Colorado, Massachusetts, and the Federally Facilitated Marketplace (7, 47, 48, 101, 102).^¶¶^

Based on this evidence, CBO concludes that the expansion of zero-premium plans in the marketplace increased take-up by a moderate amount. These factors were included in CBO’s estimate of the effects of several health coverage policies in 2025 (103).

## Opportunities for Future Research

In this section, we highlight three opportunities for future research that address different limitations of the existing literature. First, some important topics have been understudied, for example the role of brokers in the ACA marketplaces. Second, the research lacks common, foundational measures of program eligibility. We call for an open-source, comprehensive microsimulation of Medicaid and marketplace eligibility. Such measures would make it easier for researchers to argue that their research captures the effects of nonfinancial factors on take-up rather than mismeasurement or changes in program eligibility. Third, the external validity of research conducted in one domain, program, or state is often unclear. Additional discussion of external validity or research that bridged topics would allow research to have wider impact and reduce CBO’s need to extrapolate. We provide the example of research on the relative take-up of employment-based insurance and nongroup insurance. However, we also note that research based on one or two states (for example, the implementation of work requirements for Medicaid in Arkansas and Georgia) requires CBO and policy analysts at other organizations to make assumptions about how to apply the results to other states. Thoughtful guidance on this subject from the authors of the work is deeply appreciated.

### Role of Brokers in Marketplace Enrollment.

In recent years, agents and brokers have come to play a dominant role in marketplace enrollment through Healthcare.gov. In part due to the introduction of the enhanced direct enrollment platforms that allow agents and brokers to enroll consumers without visiting HealthCare.gov, the share of active enrollments (as opposed to automatic reenrollments) facilitated by agents and brokers climbed to 70% by 2023.^##^ Agents and brokers facilitate enrollment and retention by guiding enrollees through potentially complicated enrollment and verification processes. At the same time, agents and brokers may steer consumers toward suboptimal plans, and some have fraudulently enrolled consumers or switched them into plans they did not desire.

We concur that more research is needed on the effect of agent or broker assistance on the take-up and retention of marketplace insurance and plan selection (104). Questions with important budgetary considerations include the following:

- What effect do brokers have on enrollment, effectuation, and reenrollment rates under current law? What role do they play in mitigating the effects of administrative burdens and issues related to choice infrastructure on enrollment? How have these effects differed over time and across states due to policy?
- How do the plan selections of enrollees assisted by agents and brokers differ from unassisted enrollees and those assisted by federally funded Navigators? Are they more or less likely to select dominated plans or bronze plans over CSR silver plans or off-marketplace over on-marketplace plans?
- Are enrollees who are assisted by agents and brokers more likely to have a discrepancy between their advanced premium tax credit and premium tax credit at the time of reconciliation? I.e., do agents and brokers coach consumers to report incomes that maximize their marketplace subsidies?

The Centers for Medicare and Medicaid Services and state-based marketplaces can support research into these questions by sharing data on agent- and broker-assisted enrollment.

### Imputed Medicaid and Premium Tax Credit Eligibility.

Research on the take-up of Medicaid and premium tax credits would greatly benefit from the existence of a public microsimulation model of eligibility for those programs along the lines of NBER’s TAXSIM model. Survey and administrative data report enrollment figures but not eligibility, which must be imputed by researchers. Furthermore, while the eligibility criteria for Medicaid and PTCs are quite complicated, researchers—who have limited time and resources—generally base eligibility imputations only on age, income, reported coverage, and factors that are particularly salient to the projects they are working on (CBO and analysts at institutions like RAND and the Urban Institute have imputed many of them for their microsimulation models. However, these imputations are not publicly available, either because they incorporate confidential data or are proprietary.). Some recent work has provided updates to the estimates on take-up for Medicaid and other insurance types, but a collaborative, open-source model would provide even greater value to the research community (6).

Imputing eligibility for Medicaid and PTCs jointly is far superior to imputing either in isolation. While eligibility for Medicaid and PTCs is largely determined by income, many other factors come into play. Among these are immigration status, disability status, pregnancy and childbirth, and income volatility. Notably, income volatility is crucial for imputing both Medicaid and PTC eligibility as Medicaid eligibility is based on monthly income and PTC eligibility is based on estimated annual income. As a result, many people who are seemingly ineligible for Medicaid or PTCs based on retrospective annual income measures reported in survey data are in fact eligible for these programs (7, 105).

A collaboration between researchers on a public microsimulation model of Medicaid and PTC eligibility would spur additional research into the topics discussed in this article and increase research quality. Hosting the model on a platform like Github would allow for continuous maintenance and contributions from researchers who found the model lacking for the topic they study. Accessible syntax and structure and parameterization of factors like the enforcement rate of eligibility criteria would encourage use of the model.

### Nonfinancial Factors in the Relative Take-Up of Nongroup and Employer-Based Coverage.

Take-up of marketplace insurance is low relative to employer-sponsored insurance despite both being private insurance. Some of the gap can be explained by financial factors, including differences in out-of-pocket premiums, plan generosity, network breadth, and the income distribution of potential enrollees. Nonfinancial factors, including enrollment assistance and the automatic deduction of premiums from an employee’s paycheck, may help to explain the rest of the gap. Research on nonfinancial factors that differ between the markets for employer-sponsored insurance and the nongroup market would help CBO project coverage under policies that shift eligibility between employer-sponsored insurance and nongroup coverage, such as a policy that makes individual health coverage reimbursement accounts more widely available, and the effects of policies that make the nongroup market more or less similar to the market for employer-sponsored insurance.

## Conclusion

Take-up rates for Medicaid and subsidized marketplace insurance have a major effect on the federal budget and a growing body of evidence suggests that nonfinancial factors materially affect health insurance take-up rates. This article describes how CBO incorporates that evidence into its health insurance baseline and cost estimates, embedding nonfinancial effects directly into its Medicaid enrollment model and into the structure and calibration of HISIM2. Incorporating these factors within these frameworks allows the agency to capture interactions with (direct) financial incentives, heterogeneity across populations, and interactions across insurance types. Even small changes in take-up attributable to nonfinancial factors can affect the coverage of millions of people and substantially alter projected federal costs. Continued research on eligibility measurement, enrollment assistance, and behavioral responses to policy changes would further improve the accuracy of coverage projections and cost estimates.

## Data Availability

There are no data underlying this work.

## Footnotes

^*^All proposals are estimated relative to the agency’s baseline projections for coverage and budgetary outcomes over a 10-year period.

^†^Nongroup insurance is private insurance that is purchased through the health insurance marketplaces established under the Affordable Care Act (ACA) or outside them, through brokers or directly from insurers.

^‡^For more information about HISIM2, see (106, 107).

^§^In reviewing this literature, CBO makes no judgement on whether the changes in take-up rates induced by policies are justified.

^¶^The premium tax credit is an advanceable and refundable credit that reduces enrollees’ out-of-pocket costs for the premiums they pay for health insurance obtained through the marketplaces.

^#^In the context of HISIM2, households refer to “health insurance units,” (HIUs) which are defined in the model as groups of people who live together and would be eligible for coverage under the same employment-based family plan. Asingle person is his or her own HIU. Otherwise, an HIU is the set of individuals who could be covered by a family plan—that is, a plan that covers an employee and his or her dependents—if an employer offered that plan.

^‖^For details on the specification of the utility function in HISIM2, see (107).

^**^For marketplace coverage, federal subsidies come in the form of the premium tax credit (PTC)—a tax credit that eligible people can use to lower the out-of-pocket cost of their monthly premiums for health insurance coverage purchased through the ACA marketplaces. Some marketplace enrollees have access to “zero-premium plans” for which the PTC fully covers the premium.

^††^Between March 2020 and March 2023, all states provided continuous eligibility to enrollees in Medicaid and Medicaid expansions of CHIP as a condition to receive additional federal Medicaid funding. This provision allowed people to remain enrolled during that period regardless of changes in their eligibility.

^‡‡^Under the ACA, marketplace enrollees with income below 250 percent of the FPL are eligible but not required to enroll in plans with reduced cost-sharing.

^§§^In addition to PTCs, marketplace enrollees may also be eligible for cost-sharing reductions (CSRs), which reduce patients out-of-pocket costs for health care but are available only in silver-tier plans. In 2017, federal payments to insurers to fund CSRs were discontinued. Insurers in most states recouped this lost funding by increasing premiums for silver plans; this practice is known as “silver loading.” Because PTCs under the ACA are tied to the price of a benchmark silver plan, silver loading increased the dollar value of PTCs. For many low-income enrollees, the larger PTCs fully covered the premiums of less expensive plans.

^¶¶^Under the ACA, states have the option to run a state-based marketplace that manages eligibility determinations, applications, and enrollment. For all other states, these functions are managed through the “federally facilitated marketplace” run by the Department of Health and Human Services.

^##^Calculation: Numerator from (108) and denominator from (109).
